# First report of the giant liver fluke (*Fascioloides magna*) in Slovenia and molecular species confirmation based on the ITS2 marker

**DOI:** 10.3389/fvets.2025.1673629

**Published:** 2025-10-03

**Authors:** Diana Žele Vengušt, Darja Kušar, Gorazd Vengušt, Petra Bandelj

**Affiliations:** ^1^Institute of Pathology, Wild Animals, Fish, and Bees, Veterinary Faculty, University of Ljubljana, Ljubljana, Slovenia; ^2^Institute of Microbiology and Parasitology, Veterinary Faculty, University of Ljubljana, Ljubljana, Slovenia

**Keywords:** giant liver fluke (*Fascioloides magna*), red deer (*Cervus elaphus*), fallow deer (*Dama dama*), roe deer (*Capreolus capreolus*), PCR, Sanger sequencing

## Abstract

**Introduction:**

The giant liver fluke, *Fascioloides magna*, is a parasite that primarily infects wild and domestic ruminants. Originally from North America, it has been an invasive species in Europe since the nineteenth century. Of the three natural foci that have become established in Europe, the Danube floodplain forest is the one that is still spreading. The first outbreak of fascioloidosis in Slovenia described in this report indicates that red deer (*Cervus elaphus*), fallow deer (*Dama dama*), and roe deer (*Capreolus capreolus*) are the three wild ruminants affected by the parasite.

**Materials and methods:**

A total of 22 frozen deer livers (14 red deer, five fallow deer, and three roe deer) were subjected to necropsy, parasitological and molecular examinations. Molecular detection of the extracted parasites was performed using species-specific PCR and Sanger sequencing techniques.

**Results:**

The presence of *F. magna* was confirmed in all liver samples. The infected livers of red deer and fallow deer were characterized by marked enlargement and the development of pseudocysts and distinct black pigmented lines within the parenchyma, whereas the livers of roe deer were of normal size and contained only migratory paths.

**Discussion:**

The first report and molecular confirmation of *F. magna* in red deer, fallow deer, and roe deer in Slovenia indicate a northward spread of the trematode along the eastern side of the Mura River. Hunters, veterinarians, and farmers should be made aware of the existence of *F. magna* in north-east Slovenia and encouraged to report any liver abnormalities in ruminants. Future research should aim to investigate the spread of *F. magna*, its origins and economic impact.

## 1 Introduction

*Fascioloides magna*, commonly known as the giant liver fluke or American liver fluke, is a liver-inhabiting parasite that primarily infects both wild and domestic ruminants. Originally from North America, *F. magna* is recognized as an invasive species in Europe (1). Typical for the Fasciolidae family, the life cycle of *F. magna* includes a wild ruminant as the definitive host, while pulmonate freshwater snail species belonging to the Lymnaeidae family serve as an intermediate host (2). Its most common definitive hosts in North America are caribou (*Rangifer tarandus*), mule deer (*Odocoileus hemionus hemionus*), black-tailed deer (*Odocoileus hemionus columbianus*), white-tailed deer (*Odocoileus virginianus*) and wapiti (*Cervus elaphus canadensis*) (3). In Europe, *F. magna* primarily infects cervids (family Cervidae), which serve as definitive hosts; more rarely, the parasite occurs in other ruminants as aberrant hosts, and occasionally in suids, horses and rodents as dead-end hosts (1). According to the currently accepted terminology specified by Pybus (3), for example, red deer (*Cervus elaphus*), fallow deer (*Dama dama*) and white-tailed deer are categorized as definitive hosts, wild boar (*Sus scrofa*) as a dead-end host and roe deer (*Capreolus capreolus*) as an aberrant host of *F. magna* (1, 4–6).

From the North American continent, *F. magna* was introduced to Europe on at least two occasions by various non-native deer species, including the white-tailed deer and the wapiti (7). Since its first appearance in the 19th century, three natural focal points have been established in Europe: La Mandria Regional Park in northern Italy, an area spanning the Czech Republic and south-western Poland, and the Danube floodplain forests (7). The latter include parts of Austria, Croatia, Hungary, Slovakia, Serbia (4), and Romania (8). The Danube floodplain forests appear to be the only area in Europe where *F. magna* is still spreading, which may be partly due to the abundance of its intermediate host, the snail *Galba truncatula* (syn. *Lymnaea truncatula*) (4).

The migration of the immature flukes through the liver parenchyma of the definitive host leads to the formation of tunnels or triggers the development of pseudocysts, potentially resulting in the rupture of the liver parenchyma, fibrosis and cirrhotic changes, while the eggs produced by the parasite can obstruct the bile ducts; infection with the fluke also leads to severe and permanent lesions on the liver surface (1, 3, 9). As a result, the overall metabolic processes and performance of the host organism are impaired (4). Within the Cervidae family in Europe, both red deer and fallow deer can harbor large infrapopulations of the liver flukes [i.e., show high intensities of infection with *F. magna*; (10)], which are associated with extensive damage to liver tissue although they often show no clinical signs (11). In contrast, fatal cases have been reported in roe deer, domestic sheep (*Ovis aries*) and goats (*Capra hircus*) (11). However, recent reports indicate that roe deer can survive infection with *F. magna* and can be classified as a competent definitive host, suggesting possible adaptive processes in the host–parasite interaction (1, 9, 12–15); the latter was suggested to affect more the parasite than the host, namely that *F. magna* decreased its pathogenicity in the case of roe deer to avoid loss due to killing the host (1, 14). Parasite transmission from wild to domestic ruminants generally occurs where these species graze together (16–18).

Despite the publication of numerous studies on the infection of wild ruminants with *F. magna* in countries neighboring Slovenia, including Croatia, Austria, Hungary, and Italy, in the last two decades (6, 13, 14, 19–21), there has been no report for Slovenia to date. Slovenian professional game wardens have recently detected unusually extensive changes in the livers of red deer, roe deer, and fallow deer and have sent samples for examination. The aim of this study was to investigate the causes of the changes observed in the livers and to assess the extent of the lesions attributed to the giant liver fluke in three deer species in Slovenia as well as to confirm its identity by molecular methods (PCR and Sanger sequencing). This is the first report on the occurrence of *F. magna* in the territory of Slovenia.

## 2 Materials and methods

### 2.1 Infected animals and sampling

**Figure 1:**
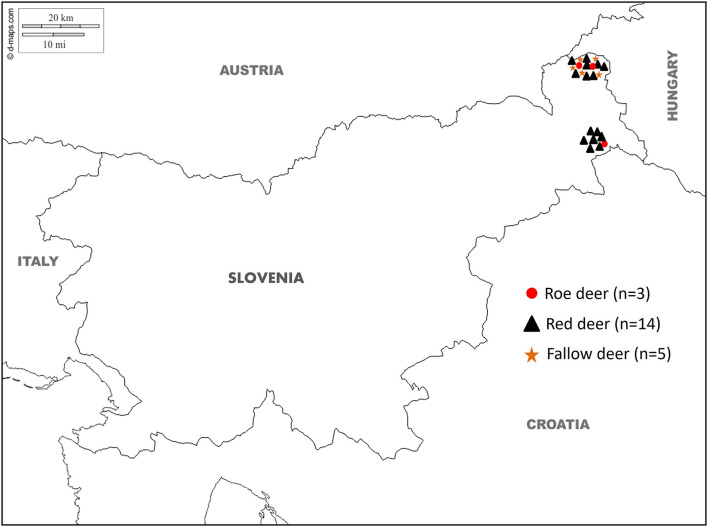
Map showing the geographical location of the special purpose state hunting grounds (SPHG) in the Prekmurje region (SPHG Fazan Beltinci and SPHG Kompas Peskovci) with the site where the liver samples collected from roe deer (*Capreolus capreolus*; red dot), red deer (*Cervus elaphus*; black triangle) and fallow deer (*Dama dama*; orange star) tested positive for *Fascioloides magna*.

During the summer and winter of 2024, professional gamekeepers of the special purpose state hunting grounds (SPHG) in the Prekmurje region (SPHG Fazan Beltinci and SPHG Kompas Peskovci) in north-eastern Slovenia (Figure 1) observed unusual changes in the livers of 22 animals when they dressed the animals after the regular annual culling. According to the observations of gamekeepers, only minor weight loss was observed in some animals. The documented lesions on the livers were described as cyst-like, pus-filled formations distributed throughout the parenchyma, exhibiting a fragile consistency. A total of 22 frozen deer livers (14 red deer, five fallow deer, and three roe deer) were provided by the Veterinary Hygiene Services to the Veterinary Faculty, University of Ljubljana, where they were subjected to post-mortem examination and parasitological analysis followed by molecular species confirmation of the extracted parasites. The age of the animals was subsequently estimated by an authorized committee of hunters during the obligatory annual inspection of hunted ungulates (22), where an estimate on the eruption patterns and tooth wear was made. The age of the animals in the study ranged from 5 months to 10 years, with an average age of 3 years. The approval of the Ethics Committee/Welfare Authority was not required as all samples were collected post-mortem.

### 2.2 Post-mortem liver examination

The livers of the deer were examined macroscopically for shape, size, fibrin deposits, irregular formations and the presence of stripes with black pigmentation (traces of iron porphyrin pigments). Each liver was photographed from both sides and then cut into 1 cm thick slices. Gross lesions and fluke-like parasites were classified according to their characteristics, including the presence of juvenile/adult flukes, active or degrading pseudocysts and migratory paths of the flukes. Livers were classified as acutely infected if they had traces of iron porphyrin and the presence of juvenile flukes with their migratory paths, whereas chronically infected livers contained both juvenile and adult flukes with their migratory paths and active or degraded pseudocysts. The parasites collected at post-mortem liver examination were preserved in 70% ethanol.

### 2.3 Parasitological analysis

The fluke-like parasites were examined under a light microscope. Samples of livers were also used to find eggs using the sedimentation method after the livers had been thoroughly washed in a bucket of water.

### 2.4 Molecular species determination

After morphological examination of the fluke-like parasites, molecular methods were used to confirm the parasite species. Samples (0.5 × 1 cm) of five parasites collected from four animals (three red deer and one roe deer) were subjected to DNA extraction using the iHelix kit (Institute of Metagenomic and Microbial Technologies, Slovenia; https://www.ihelix.eu/, accessed on 22 July 2025) according to the manufacturer's instructions. The extraction protocol included mechanical shearing with bead-beating for 45 s at 6,400 rpm three times (MagNA Lyser Instrument; Roche, Switzerland), and a combined enzymatic/heat induced lysis. After washing, 100 μl of DNA was eluted and stored at −20 °C until further analysis.

For species determination, three PCRs and Sanger sequencing were employed, targeting the ribosomal ITS2 region (23–25). Each primer pair [FH_ITS_SPEC_F / FH_ITS_SPEC_R targeting *Fasciola hepatica* (23), FM_ITS_SPEC_F / FM_ITS_SPEC_R targeting *F. magna* (23) and FAS_uni1/FAS_uni2 targeting both species (25)] was used in a separate 25-μl PCR reaction mixture, which contained 2.5 μl of the extracted DNA, 0.5 U of Platinum Taq DNA Polymerase (Invitrogen by Thermo Fisher Scientific, Waltham, MA, USA), 2.5 mM MgCl_2_ and 1 × PCR buffer supplied by the manufacturer, 1 μM of each primer and 0.25 mM of each dNTP (Applied Biosystems by Thermo Fisher Scientific). Amplification was performed in the VeritiPro Thermal Cycler (Applied Biosystems by Thermo Fisher Scientific) according to the published protocols (25); for the species-specific PCRs, the protocol consisted of initial denaturation at 94 °C for 5 min, 30 cycles of denaturation at 94 °C for 1 min, annealing at 51 °C for 1 min and extension at 72 °C for 2 min, and final extension at 72 °C for 10 min. The obtained PCR amplicons with the expected length of 152 bp for *F. magna* and 112 bp for *F. hepatica* (25) were analyzed with the QIAxcel capillary electrophoresis system (Qiagen, Germany) using the QIAxcel DNA High Resolution Kit, QX Alignment Marker 15–1,000 bp, QX Size Marker 50–800 bp, OM500 separation method and a sample injection time of 10 s according to the manufacturer's instructions.

For additional species confirmation, PCR employing the universal primers FAS_uni1 and FAS_uni2 was used (25) and the obtained amplicons were subjected to Sanger sequencing. The amplification protocol consisted of initial denaturation at 94 °C for 3 min, 30 cycles of denaturation at 94 °C for 30 s, annealing at 54 °C for 30 s and extension at 72 °C for 30 s, and final extension at 72 °C for 10 min. The obtained amplicons were analyzed with the QIAxcel capillary electrophoresis system (Qiagen, Germany) as described above and sequenced in both directions (Eurofins Genomics Europe, Germany). The retrieved sequence fragments were imported into Geneious Prime v2022.1.1 (Biomatters, New Zealand). Sequence ends were quality trimmed from chromatograms in the Sequence View tab and pairwise alignment of the two corresponding sequences was performed using default Geneious Alignment parameters (alignment type: global alignment with free end gaps, cost matrix: 65% similarity, gap open penalty: 12 and gap extension penalty: 3).

The obtained sequences were subjected to blast search (https://blast.ncbi.nlm.nih.gov/; accessed on 29 August 2024) of the core nucleotide database. To confirm the results of blast search, the constructed sequences (*n* = 5) were supplemented with six *F. magna*, six *F. hepatica*, three *Dicrocoelium dendriticum*, and three *Paramphistomum cervi* GenBank hits comprising complete ITS2 sequences to construct the phylogenetic tree; the maximum likelihood tree with Tamura-Nei model (26) was constructed in MEGA11 (27) with default parameters (rates among sites: uniform rates, gaps/missing data treatment: use all sites, ML heuristic method: nearest-neighbor-interchange, initial tree for ML: make initial tree automatically – NJ/BioNH).

## 3 Results

### 3.1 Post-mortem liver examination findings

**Figure 2:**
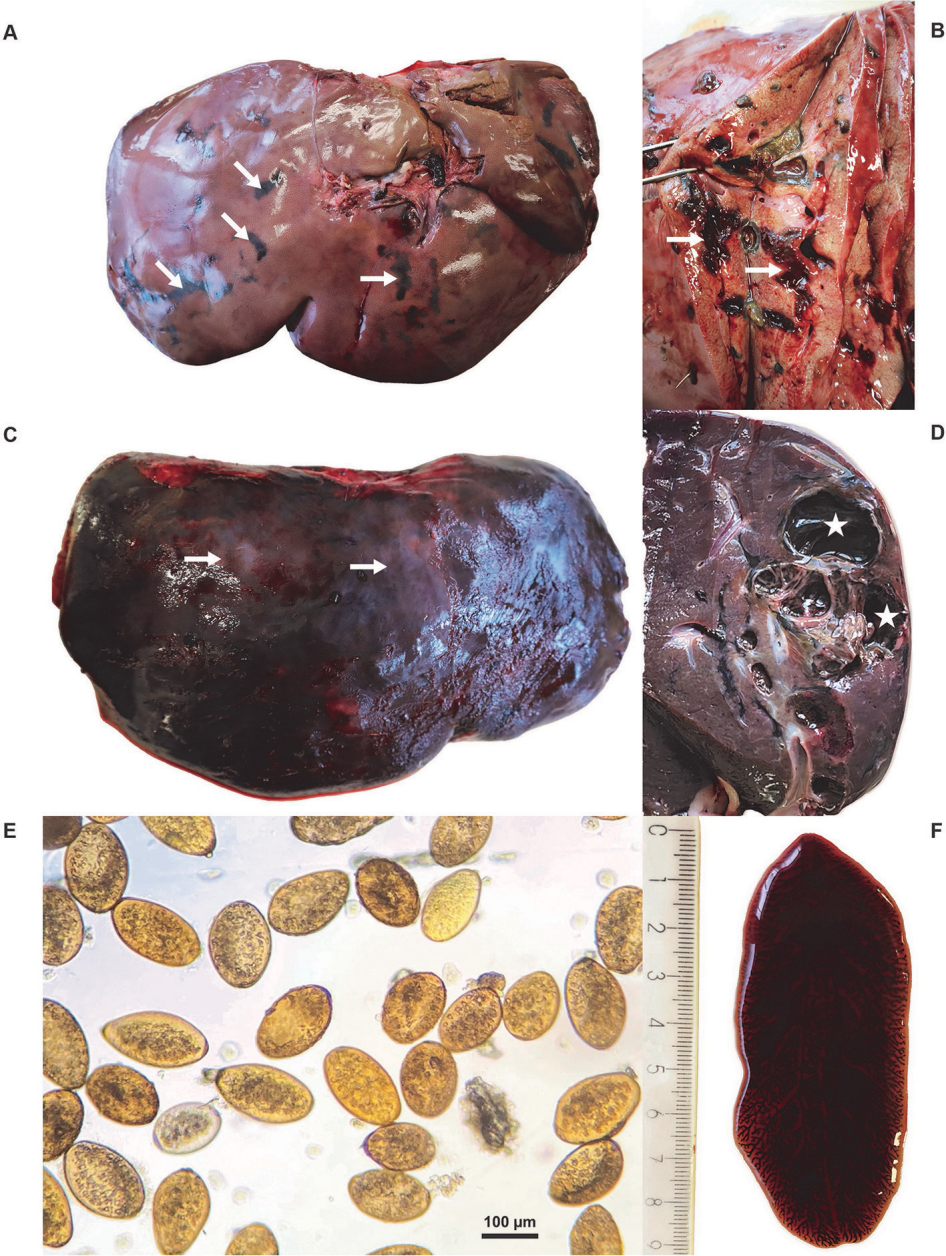
**(A)** Macroscopic lesions of an infected red deer (*Cervus elaphus*) liver with typical traces of iron porphyrin pigments (white arrows). **(B)** Cross-section of the liver of a red deer with traces of migration (white arrows) and visible yellow detritus in the parenchyma. **(C)** The irregular surface of the red deer liver with nodular protrusions (white arrows) evident through the capsule and fibrin deposits. **(D)** The cross-section of the red deer liver with pseudocyst (white stars). **(E)** Eggs of *Fascioloides magna*. **(F)** Adult *F. magna*, isolated from a pseudocyst of the liver parenchyma.

In gross pathology, the livers of 11/14 red deer and 4/5 fallow deer were markedly enlarged and the lesions generally involved more than 75% of the organ; the livers of 3/14 red deer and 1/5 fallow deer were of normal size. Irregular bands of black pigmentation (i.e., traces of iron porphyrin pigments) were noted on the surface and in the parenchyma of most livers (Figure 2A); black pigmentation of the liver is considered pathognomonic for an infection with *F. magna* (1). On a cross section of the liver, the traces of migration and yellow detritus in the parenchyma were visible (Figure 2B). The tissue lesions were distributed throughout the liver parenchyma and consisted of migratory fluke paths (Figures 2A, B), and multiple, firm and thick-walled fibrous pseudocysts with a diameter of 1–6 cm (Figures 2C, D). The livers of the 3/3 roe deer were of normal size and contained adult flukes and their migratory paths. Excessive hemorrhages with multiple migration paths were observed on the cross-section of most livers of all tree species. From the pseudocysts and migratory paths in the livers of red deer, fallow deer and roe deer, 1–51 (median 5.5), 1–17 (median 3), and 2–8 (median 6) leaf-shaped parasites were extracted, respectively. The extent of liver damage varied, likely depending on the intensity (i.e., number of flukes per liver) and/or duration of infection (28). Chronic infection was confirmed in 11/14 red deer and 4/5 fallow deer. Less severe changes in the liver tissue were observed in 3/14 red deer and 1/5 fallow deer aged less than 1 year. Roe deer included in the study were adults, and while their liver damage was severe, pseudocysts were absent.

### 3.2. Parasitological findings

After sedimentation, operculate yellow eggs, 110–140 × 70–80 μm in size, were recovered from the livers of deer (Figure 2E). The eggs were found in 11/14 red deer, 5/5 fallow deer and 2/3 roe deer liver samples. Under the light microscope, the shape and morphological features of the parasites, recovered from the livers of all deer submitted to the study, were typical of flukes from the family Fasciolidae (Figure 2F); the parasites were dorsoventrally flattened with a leaf-like shape, the oral and ventral suckers located in the anterior third of the parasites, and branched intestinal caeca. The parasites measured 1.7–8.5 cm, 1.5–8 cm, and 1.3–7.7 cm in length in red deer, fallow deer and roe deer, respectively, while their width ranged from 1 to 3.5 cm in all three host species.

### 3.3 Molecular findings

**Figure 3:**
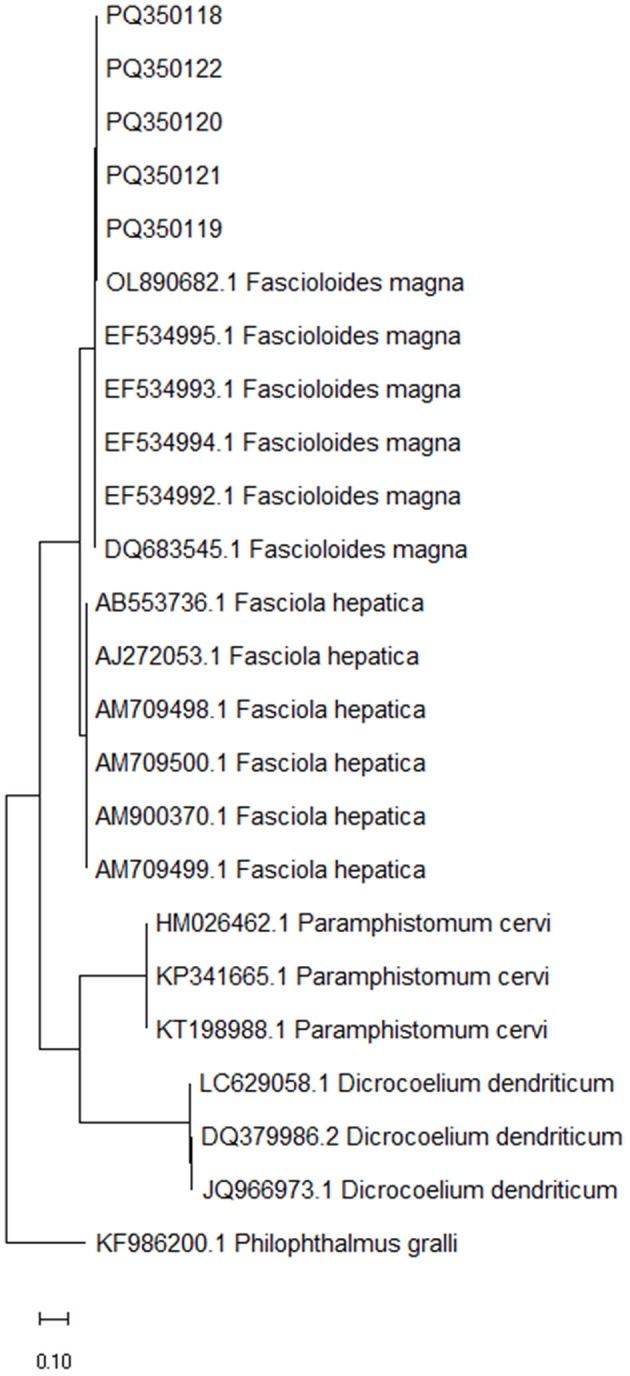
Maximum likelihood phylogenetic tree of ITS2 sequences from *Fascioloides magna, Fasciola hepatica, Paramphistomum cervi* and *Dicrocoelium* dendriticum, representing the liver and stomach flukes of domestic and free-living ruminants (24). Sequences obtained in the present study (PQ350118, PQ350119, PQ350120, PQ350121, and PQ350122) are clearly clustering among the rest of *F. magna* sequences. GenBank accession numbers are listed in addition to taxonomic species. The tree is drawn to scale, with branch lengths measured in the number of substitutions per site. ITS2 sequence from *Philophthalmus gralli* (GenBank accession no. KF986200.1) was used as an outgroup to root the tree.

With the species-specific PCRs, all samples of fluke-like parasites generated a 152-bp band characteristic of *F. magna* and were negative for *F. hepatica* (no characteristic 112-bp band was present), suggesting the parasitic hepatitis of the inspected animals due to *F. magna* (fascioloidosis). With the universal PCR, all samples generated the expected band of 358 bp. After Sanger sequencing, quality trimming and pairwise alignment of each pair of the corresponding sequences, alignment of all five constructed consensus sequences (submitted to GenBank under the accession numbers PQ350118–PQ350122) was performed and showed that all inspected fluke-like samples contained an identical ITS2 sequence with no polymorphisms observed. According to blast search, the consensus sequences were most similar to *F. magna* (99.70%−100% identity with 95%−100% query cover); other blast hits belonged to *Fasciola* species and with much lower identity (< 91.95%). The constructed phylogenetic tree, comparing the five consensus sequences and ITS2 sequences of several fluke species (*F. magna, F. hepatica, D. dendriticum* and *P. cervi*), showed a clear clustering according to the species, placing the five sequences obtained in this study into *F. magna* cluster (Figure 3).

## 4 Discussion

For over two decades, Slovenia has been surrounded by neighboring countries where infection with *F. magna* has been confirmed in wildlife (6, 13, 14, 19–21). In this study, we combined gross pathological and parasitological examinations with molecular analysis to confirm the presence of *F. magna* in Slovenian deer. For *F. magna*, PCR and sequencing are particularly useful when eggs detected using coproscopy or larval stages of flukes are subjected to species identification as these are difficult to distinguish morphologically, especially when there is a co-infection with developmental stages of various flukes, which induce similar clinical signs, or when homogenized liver samples or snail intermediate hosts are analyzed (23–25). In adult specimens, molecular approach is the ultimate method for species confirmation, complementing gross pathology and parasitology. The ITS2 sequences obtained here expand the available genetic database for *F. magna*, improving the reliability of future blast searches. This study provides the first documented and molecularly confirmed report of *F. magna* in Slovenia, establishing a foundation for future research on its distribution, transmission and epidemiology in the region.

In Europe, *F. magna* is considered an invasive species (4) with a high potential to spread and colonize new geographical territories and establish local subpopulations (6). Although infection of humans has not been documented (6), the zoonotic significance of *F. magna* remains unclear, particularly in light of a recent report of an infected primate (29). Wild ungulates in Europe such as red deer and fallow deer are generally categorized as definitive hosts, while roe deer used to be considered aberrant hosts but their assessment has been varying recently (1, 9, 13, 14). Significant differences in clinical symptoms and necropsy findings are generally observed between these species (4). Definitive hosts rarely show clinical signs and typically survive the infection, but they contribute to the spread of the parasite in the environment. In roe deer, however, the disease usually progresses rapidly and often leads to death, as the liver parenchyma or other organs are severely destroyed by the migration of flukes due to the failure in pseudocyst formation (30, 31). However, Konjević et al. (13) discovered pseudocysts in 7/34 inspected roe deer livers, suggesting chronic infection with prolonged survival; pseudocyst development in the infected roe deer was also reported by other studies (12, 14, 15, 25). Although there was no pseudocyst formation in roe deer, two roe deer livers were positive for fluke eggs in our study. Egg production and egg sheading in roe deer have been reported as a common phenomenon in endemic areas of Europe (1, 9, 13, 14).

In the present study, samples were selected by professional game wardens based on visible liver abnormalities observed during the evisceration of various deer species, which is a highly sensitive and specific method for monitoring populations that may be infected for the first time (14). The surface of the livers in this study appeared irregular with nodular protrusions, whereas in the parenchyma, areas of dark pigmentation, hemorrhages, and pseudocysts (except in roe deer) filled with brown mucous fluid and adult flukes were observed. Flukes were also found within the migratory tracts in the liver parenchyma. The lesions of the liver parenchyma are usually caused by the limited migration of immature flukes, followed by encapsulation of the mature specimens (3). Most of the examined livers of red deer and fallow deer were significantly enlarged, showing moderate to severe tissue damage, whereas the livers of roe deer were of normal size with but contained severe lesions. Livers of the inspected animals contained a median of 5.5 flukes in red deer, three in fallow deer and six in roe deer, indicating a generally low intensity of infection in the studied population. As already reported by Sommer et al. (28), the extent of liver damage in the infected animals was related to both the number of flukes and the duration of infection. Although no pseudocysts were observed in roe deer, fluke eggs were recovered form 2/3 livers, indicating completion of the fluke life cycle. This may suggest a potential adaptation trend in the host-parasite interaction, as previously described in e.g., the neighboring Croatia (13, 15). However, it should also be noted that only three roe deer with characteristic liver lesions were subjected to examination in our study. It can be assumed that in Slovenia the infection period is yet too short to enable a full establishment of the adaptation process in roe deer.

To accurately assess the prevalence of *F. magna* and the factors associated with its occurrence, it is essential to examine nearly the entire annual hunting bag of red deer, fallow deer and roe deer in Slovenia for the presence of liver flukes. In the neighboring Hungary, however, the endemic area of *F. magna* is continuously expanding (14). The last reported occurrence of the parasite near the Slovenian border was in 2018 in the Hungarian region of Transdanubia, in the south-western part of Hungary (14, 21). Given the ongoing expansion of the endemic area (14), it is assumed that *F. magna* has migrated north of the Mura River and infected wild ruminants east of the river, close to the Hungarian border. In the present study, all infected deer originated only a few kilometers from the known Hungarian locations. This confirms the spread of *F. magna* beyond the Danube floodplain forests and supports the hypothesis of its migration. These findings underscore the need for continuous surveillance north of the most recently reported epidemiological foci in Transdanubia (14). Furthermore, the parasite has recently been detected also in Serbia, along the banks of the Sava River (32), which, like the Mura, flows into the Danube. As the Sava also flows through Slovenia, it could represent another potential northern entry route for the parasite into the country. In the Hungarian study from 2018 (21), the authors assume that the spread of *F. magna* is driven by both natural dispersion and human-influenced factors.

To assess the transmission routes or introduction events and the intraspecific genetic diversity of *F. magna*, genetic typing should be performed; this is usually based on the cytochrome oxidase subunit I (*cox1*) and nicotinamide dehydrogenase subunit I (*nad1*) mitochondrial genes (7, 33, 34). In Austria, for example, all the analyzed samples were of the same haplotype, indicating a single introduction event, bottleneck effect and/or genetic drift (33). In our study, PCR and sequencing of the ITS2 region were performed, aiming at molecular confirmation of the species. As *cox1* and *nad1* sequencing was not conducted, comparison with *F. magna* from neighboring countries is currently not possible. The obtained ITS2 sequences showed no genetic heterogeneity, which was confirmed before (23). Also, when a phylogenetic tree was constructed using all available full-length ITS2 sequences from *F. magna* (*n* = 20; GenBank accessed on 27 August 2025) originating from various geographic locations (Austria, Canada, Czech Republic, Romania, Slovakia, United States, and Slovenia), almost no genetic variation was observed (data not shown).

While surveillance of the disease, its spread and its impact on wild and domestic ruminant populations is urgently needed, the question is what options are available to limit the spread of the fluke and its economic impact. Routine antiparasitic treatment in natural environments is generally not advisable due to the complexity of ecological interactions and concerns about drug resistance; it should only be applied in well-justified, exceptional cases (35). For animals kept in farms and game parks, antiparasitic treatment is a viable option as it allows for the treatment of all animals and the evaluation of therapeutic efficacy (35, 36). In addition, control of the intermediate host (snails) has been shown to be an effective method for eradicating fascioloidosis in endemic areas, while the development of a vaccine against *F. magna* infection is a major challenge due to the various life stages of the parasite that need to be controlled in the host (37, 38). The timing of antiparasitic treatment can be strategically scheduled to begin in the winter months, when the risk of re-infection is significantly lower (6, 35, 39). In contrast, the surveillance and control of fascioloidosis in domestic animals should be based on targeted selective treatment following coprological analysis (40). In competent hosts, *intra-vitam* diagnosis of active infection by egg coproscopy may serve as a useful adjunct to disease surveillance (25), particularly as there are no coproantigen tests commercially available (40). However, domestic ruminants are not competent hosts and coproscopy would result in very low sensitivity. On the other hand, co-grazing of livestock and wild cervids has been shown to have a diluting effect on the prevalence and infection intensity of *F. magna* in cervids (18). The “dilution effect” hypothesis suggests that non-competent hosts ingest infective stages, thereby removing them from the pasture and interrupting their life cycle. This reduction in infective stages can lower the risk of infection, as well as the prevalence and intensity of parasitic infection in competent hosts (18, 41). In wild ruminant populations that serve as competent hosts of the giant liver fluke, egg coproscopy would be useful only during periods when hunting is banned, due to its lower sensitivity compared with post-mortem liver examination, where also prepatent infections can be detected (42). This was also the case in our study as eggs were detected in 82% of infected livers.

In conclusion, the epidemiological role of the Danube, Drava and Sava rivers in the spread of the giant liver fluke is well-known (21, 32). The first report and molecular confirmation of *F. magna* in red deer, fallow deer and roe deer in Slovenia suggest a northward spread of the trematode along the eastern side of the Mura River within just 5 years of its initial detection in south-western Hungary (21). Consequently, new epidemiological foci may emerge in the neighboring countries in the near future, which is why cooperation between countries is necessary. It is important that hunters remain aware of the risk of *F. magna* infection in the floodplain forests along other rivers flowing into the Danube. Veterinarians and livestock farmers in the north-eastern Slovenia should also consider *F. magna* as a potential cause of disease and reduced performance in cattle and small ruminants, although co-grazing of wild and domestic ruminants may be advisable to potentially reduce the parasite load (18). To date, this study cannot be regarded as an objective indicator of disease-related changes in the livers of different deer species in Slovenia, as the sampling and infection period were too short. It is likely that additional positive cases occur in the natural environment but remain undetected, as sample collection depends on the hunter's decision. In this context, passive (post-mortem examination of carcasses) and active (coprological examination of fecal samples) surveillance of wildlife and domestic livestock can provide valuable information. Health monitoring and surveillance are essential components of wildlife disease detection and management in Slovenia. Volunteer hunters and professional gamekeepers nationwide are encouraged to submit samples to the Veterinary Faculty for diagnostic evaluation, including for fascioloidosis. However, effective disease control requires close communication among veterinary authorities, hunters, farmers, field veterinarians, and wildlife disease specialists. Furthermore, future studies should not only investigate the prevalence of *F. magna* in competent or dead-end hosts, but also assess the geographical distribution, origin and economic impact of the giant liver fluke.

## Data Availability

The original contributions presented in the study are publicly available. This data can be found at the National Center for Biotechnology Information (NCBI) using accession numbers PQ350118–PQ350122.
